# Altered functional connectivity of the red nucleus and substantia nigra in migraine without aura

**DOI:** 10.1186/s10194-019-1058-0

**Published:** 2019-11-11

**Authors:** Xiaobin Huang, Di Zhang, Yuchen Chen, Peng Wang, Cunnan Mao, Zhengfei Miao, Chunmei Liu, Chenjie Xu, Xinying Wu, Xindao Yin

**Affiliations:** 1 Department of Radiology, Nanjing First Hospital, Nanjing Medical University, No.68, Changle Road, Nanjing, 210006 Jiangsu Province China; 2 Department of Neurology, Nanjing First Hospital, Nanjing Medical University, No.68, Changle Road, Nanjing, 210006 Jiangsu Province China; 3 Department of Pain Treatment, Nanjing First Hospital, Nanjing Medical University, No.68, Changle Road, Nanjing, 210006 Jiangsu Province China

**Keywords:** Migraine, Red nucleus, Substantia nigra, Functional connectivity, fMRI

## Abstract

**Background:**

Functional connectivity (FC) has been used to investigate the pathophysiology of migraine. Accumulating evidence is pointing toward malfunctioning of brainstem structures, i.e., the red nucleus (RN) and substantia nigra (SN), as an important factor in migraine without aura (MwoA). We aimed to identify atypical FC between the RN and SN and other brain areas in patients with MwoA and to explore the association between RN and SN connectivity changes and performance on neuropsychological tests in these patients.

**Methods:**

Resting-state functional magnetic resonance imaging (fMRI) data were obtained from 30 patients with MwoA and 22 age-, sex-, and years of education-matched healthy controls (HC). The FC of the brainstem structures was analyzed using a standard seed-based whole-brain correlation method. The results of the brainstem structure FC were assessed for correlations with other clinical features.

**Results:**

Patients with MwoA exhibited reduced left RN-based FC with the left middle frontal gyrus, reduced right RN-based FC with the ipsilateral superior parietal lobe, and increased FC with the ipsilateral cerebellum. Additionally, patients with MwoA demonstrated significantly decreased right SN-based FC with the right postcentral gyrus, left parietal lobule, and left superior frontal gyrus. Hypo-connectivity between the right SN and right postcentral gyrus was negatively correlated with disease duration (*r* = − 0.506, *P* = 0.004). Additionally, increased connectivity of the right RN to the ipsilateral cerebellar lobes was positively correlated with the Headache Impact Test-6 scores (*r* = 0.437, *P* = 0.016).

**Conclusions:**

The present study suggested that patients with MwoA have disruption in their RN and SN resting-state networks, which are associated with specific clinical characteristics. The changes focus on the regions associated with cognitive evaluation, multisensory integration, and modulation of perception and pain, which may be associated with migraine production, feedback, and development. Taken together, these results may improve our understanding of the neuropathological mechanism of migraine.

## Background

Migraine is the most common primary headache disorder with a reported prevalence of approximately 5.7% in men and 17.0% in women [1]. Moreover, it has socio-economic and personal consequences and was ranked as the seventh leading cause of disability worldwide in the Global Burden of Disease Survey, 2015 [2]. This primary headache disorder is often combined with neck pain, depression, and anxiety, which are also among the top 10 causes of disability worldwide, placing migraine in a central position among the world’s most disabling disorders [3]. Although a growing body of evidence supports that cortical spreading depression and the trigeminovascular system have key roles in migraine pathogenesis [4], the neuromechanism underlying migraine has not been completely elucidated and warrants further attention.

Beyond the neurovascular model, a dysfunctional neurolimbic pain network model expands on the conventional concept of migraine and it could help elucidate migraine attacks, chronicization, and refractoriness [5]. Recent evidence has suggested that the brainstem structures might be crucially involved in the generation of migraine attacks [6]. The red nucleus (RN) and the substantia nigra (SN) are two important components of the brainstem. The RN plays a critical role in motor control and it is involved in regulating muscle tension, initiating conditioned motor responses, motor learning, and nociceptive processing; and the modulation of pathological pain [7, 8]. It has been suggested that the SN plays an important role in functions such as action selection, response inhibition, and cognitive control. It is also known that several key cognitive functions are controlled by the SN such as memory, executive and visuospatial functions. A fMRI study have indiacated that the RN and SN possess close functional connections to the insular cortex and precuneus [9]. These regions are involved in the salience and default mode networks, which are primarily devoted to emotion processing, interoceptive regulation and episodic memory. Thus, when the RN and SN networks are impaired, such as in Parkinson’s Disease, affective and cognitive impairments often coexist. These cognitive functions are also often impaired in migraineurs [10]. Previous diffusion tensor imaging and constrained spherical deconvolution (CSD) studies have shown that the human RN and SN are widely interconnected with the sensorimotor and prefrontal cortices, such as the cerebellar cortex, thalamus, paracentral lobule, postcentral gyrus, precentral gyrus, superior frontal gyrus, and superior parietal lobule [11–14]. Accumulating evidence is pointing toward the RN, and possibly the SN, being involved in somatosensory processing during a migraine attack. A previous study showed that activation (hyperoxia and blood volume increase) of the RN and SN is associated with the visually-triggered symptoms of migraine [15]. A transcranial sonography study found that 70% of patients with migraine with aura (MwA) and 20% of patients with migraine without aura (MwoA) had a hyperechogenic SN [16]. In a medication-overuse headache study, functional magnetic resonance imaging (fMRI) showed reduced task-related activity in the SN of patients with medication-overuse headache compared with patients with chronic migraine [17]. The population-based MRI CAMERA study revealed increased iron concentrations in the putamen, globus pallidus, and RN of migraineurs compared with controls [18]. Despite the pathophysiologic evidence pointing toward a relation between the RN and SN and cognitive impairment, a functional imaging marker is missing, and little is known regarding the underlying structural origin of these cognitive impairments.

Resting-state fMRI (rs-fMRI) based on spontaneous blood oxygenation level-dependent (BOLD) responses has proved to be a useful noninvasive neuroimaging modality to reveal the disease-induced neural dysfunction associated with neuropathology, including alterations in the brains of migraineurs [10, 19]. Based on functional connectivity (FC) analysis, aberrant interactions of cortical networks including the default mode network, executive control network, and dorsal attention system have been previously described [20]. As a best known modulator of somatic pain transmission located in brainstem, the impaired FC of periaqueductal gray matter (PAG) networks had been reported in both animal and human headache models. Atypical FC of PAG with brain regions that are involved in nociception, somatosensory processing, emotional processing, and pain modulation was previously shown in a rat model using seed-based FC analysis [21]. Aberrant connectivity between PAG and several brain areas within nociceptive and somatosensory processing pathways were also revealed in patients with migraine during a pain-free state [22]. However, no studies have focused on the RN and SN networks. The analysis of intrinsic functional architectural changes in the RN and SN with rs-fMRI in migraineurs may have the potential to improve the understanding of disease pathophysiology.

Thus, the aim of this study was to investigate whether the RN and SN resting-state networks are disrupted in patients with MwoA compared with healthy controls and to explore the association between RN and SN connectivity changes and performance on neuropsychological tests in these patients. We hypothesized that: (1) the resting-state functional connectivity of RN and SN in patients with MwoA would be significantly different from that of HC; and that (2) these alterations would associated with specific characteristics in patients with MwoA.

## Method

### Participants

According to the International Classification of Headache Disorders, Third Edition (beta version) (ICHD-3 beta) [23], 30 patients with MwoA(4 with chronic migraine, and 26 with episodic migraine) and 22 healthy controls were recruited in the study between May 2018 and June 2019. All subjects were right-handed [24]. Migraine attacks were presented with temporal pain in 14 patients, frontal pain in 9 patients, occipital pain in 5 patients and total headache pain in 2 patients. None of the patients took any preventive medications. To avoid any possible interference from pain or pharmacological substances on BOLD signal fluctuation, patients were migraine free, without medication, for at least 3 days before the examination and were followed up 3 days after scanning to ensure that they had remained migraine free during this period. The exclusion criterias were illness affecting central nervous system function, psychotic disorder, any physical illness such as cancer, regular use or overuse of any psychoactive, preventive or chronic medication and contraindications to MRI. 22 age, sex, and years of education-matched healthy subjects were included as HC. The inclusion criterias were having no personal or family history of migraine or any other type of headaches, and free from any chronic medical condition and any chronic treatment. HC met the same exclusion criteria applied to the patient group. To minimize hormonal influences on cortical excitability, all female participants were included at mid-cycle and excluded if pregnant or breast-feeding. All participants completed the Hamilton Anxiety Scale (HAMA), Hamilton Depression Scale (HAMD), Montreal Cognitive Assessment (MoCA), Headache Impact Test-6 (HIT-6), and Migraine Disability Assessment Questionnaire. This study was approved by the Institutional Review Board of our university. All participants provided written informed consent before undergoing MR imaging.

### Imaging methods

A 3.0 T MRI scanner (Ingenia, Philips Medical Systems, Best, Netherlands) with an 8-channel head coil was used for this study. Functional images were obtained axially using a gradient echo-planar imaging sequence as follows: repetition time (TR) = 2000 ms; echo time (TE) = 30 ms; slices = 36; thickness = 4 mm; gap = 0 mm; field of view (FOV) = 240 mm × 240 mm; acquisition matrix =64 × 64; and flip angle (FA) = 90°. The fMRI sequence lasted 8 min and 8 s. Three-dimensional turbo fast-echo T1WI sequence with high resolution: TR = 8.1 mm; TE = 3.7 ms; slices =170; thickness = 1 mm; gap = 0 mm; FA = 8°; acquisition matrix = 256 × 256; FOV = 256 mm × 256 mm; fluid-attenuated inversion recovery: TR = 7000 ms; TE = 120 ms; slices = 18; slice thickness = 6 mm; gap = 1.3 mm; FA = 110°; voxel size = 0.65 × 0.95 × 6 mm^3^.

### Image processing

Imaging data analysis was performed with the Conn toolbox [25] version 18b and SPM12 (www.fil.ion.ucl.ac.uk/spm/software/spm12/) running on MATLAB R2013b (MathWorks, Natick, MA). Preprocessing of rs-fMRI images included realignment and unwarping, slice-time correction, segmentation of gray matter, white matter, and cerebrospinal fluid (CSF); normalization to the Montreal Neurological Institute template, and spatial smoothing based on a Gaussian kernel set at 8-mm full width at half maximum. The ART-based scrubbing method [26], implemented in Conn, was further used to detect outlying volumes with high motion (using a 3-mm subject motion threshold and a global signal threshold set at Z = 9). Nuisance variable regression was then performed, and the first five principal components from the segmented white matter and CSF were regressed out of the signal. The six motion realignment parameters and their first-order derivatives and outlier volumes detected in the scrubbing procedure were similarly regressed out of the signal. The data were then linearly detrended, and the residual signals were bandpass filtered at 0.01 to 0.08 Hz.

### Statistical analysis

FC between the bilateral RN and SN and the rest of the brain was tested with the seed-to-voxel approach. The seed regions of interest (ROIs) of the bilateral RN and SN were generated using the WFU PickAtlas software. Seed-to-voxel maps were computed for each subject separately based on the bilateral RN and SN seeds. For between-group comparisons, two-sample t-tests were performed to investigate differences in the FC of the bilateral RN and SN between patients with MwoA and healthy controls with a default whole-brain mask. Age and sex were included as covariates. Significance was determined at a voxel-level threshold (*P* < 0.01) with Gaussian random field theory correction at a cluster-level threshold (two-tailed, *P* < 0.05). Positive clusters based on RESTplus were generated as a binary mask, and the connective strengths of the significant regions were extracted based on the z-maps.

Differences in demographic data between patients with MwoA and healthy controls were analyzed using between-groups t-test for means and Chisquare test for proportions (*P* < 0.05 was considered significant). Regions showing significant differences between the groups were extracted to investigate the relationship between the fMRI data and clinical cognitive characteristics, disease duration and attack frequency of the patients with MwoA. Then, the mean z-values of the aberrant FC region mask were calculated within each subject. Pearson’s correlation analysis between the mean z-values and each clinical cognitive characteristic was performed using SPSS 17.0 (version 17.0; SPSS, Chicago, IL). *P* < 0.05 was considered statistically significant, corrected for age, sex, and years of education.

## Results

### Demographic data and neuropsychological tests

Table 1 summarizes the basic demographic and neuropsychological characteristics of the MwoA and healthy control groups. Neither group showed any significant difference for age (*P* = 0.122), sex (*P* = 0.094), MoCA score (*P* = 0.054), HAMA score (*P* = 0.119), HAMD score (*P* = 0.066), or years of education (*P* = 0.075).

**Table 1:** Demographic and clinical characteristics of participants

	MwoA patients(*n* = 30)	Healthy controls(*n* = 22)	*P* value
Age (years)	39.87 ± 10.43	34.27 ± 8.34	0.122
Gender (male/female)	4:26	8:14	0.094
MoCA score	25.87 ± 3.49	27.5 ± 1.97	0.054
Visuospatial and executive function	4.47 ± 0.900	4.67 ± 0.727	0.470
Name recognition	2.80 ± 0.551	2.91 ± 0.294	0.403
Attention	5.67 ± 0.711	5.82 ± 0.395	0.372
Language	2.80 ± 0.407	e2.82 ± 0.395	0.873
Abstract	1.86 ± 0.351	1.90 ± 0.403	0.736
Delayed memory	3.41 ± 1.368	3.53 ± 1.332	0.744
Orientation	5.80 ± 0.407	5.95 ± 0.213	0.111
HAMA score	40.2 ± 8.41	36.91 ± 5.72	0.119
HAMD score	40.68 ± 9.56	37.11 ± 6.09	0.066
Education (years)	13.57 ± 3.01	15 ± 2.49	0.075
Duration (years)	9.37 ± 7.77	–	–
Frequency(d/m)	5.17 ± 6.17	–	–
HIT-6 score	57.3 ± 9.27	–	–
MIDAS score	11.63 ± 8.76	–	e-

Data are presented as mean ± SD; MwoA, migraine without aura; MoCA, Montreal Cognitive Assessment; HAMA, Hamilton Anxiety Scale; HAMD, Hamilton Depression Scale; d/m, day per month; HIT-6, Headache Impact Test-6; MIDAS, Migraine Disability Assessment Questionnaire

### Comparison of FC of the ROIs

**Fig. 1:**
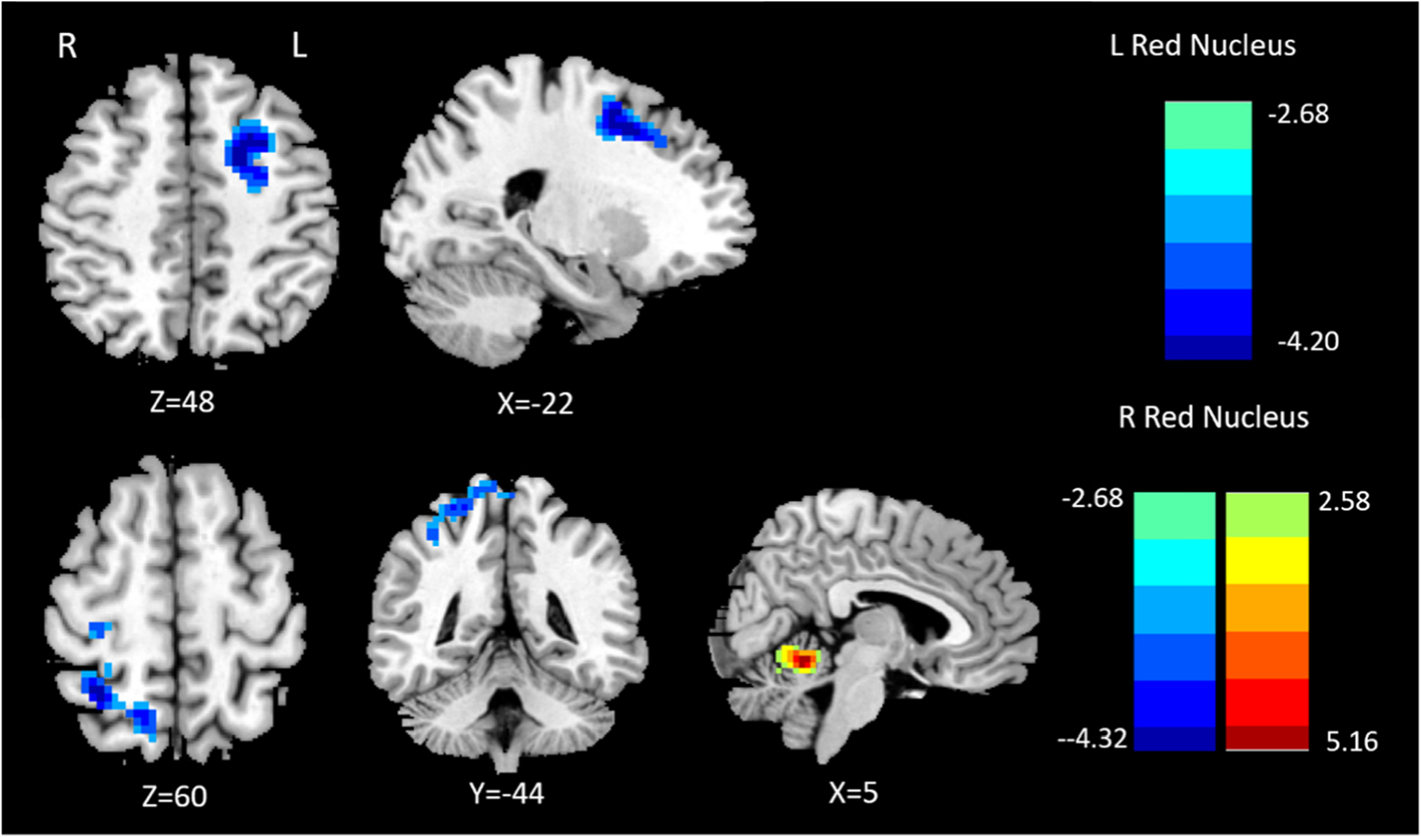
Altered effective functional connectivity between the left red nucleus and whole-brain regions in patients with migraine without aura (MwoA) compared with healthy controls (first row). Altered effective functional connectivity between the right red nucleus and whole-brain regions in patients with MwoA compared with healthy controls (second row). Blue signifies decreased functional connectivity, and red denotes increased functional connectivity

**Fig. 2:**
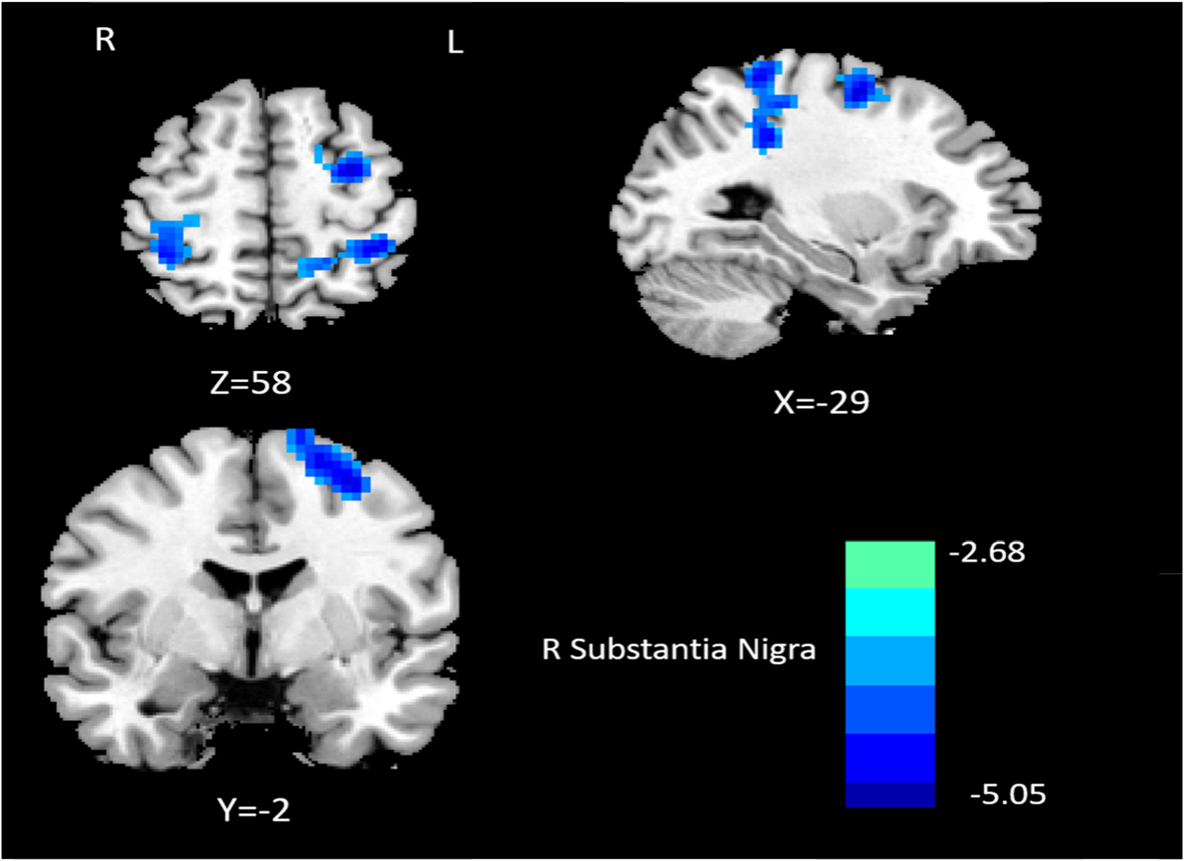
Altered effective functional connectivity between the right substantia nigra and whole-brain regions in patients with migraine without aura compared with healthy controls. Blue signifies decreased functional connectivity

Compared with healthy controls, patients with MwoA showed significantly decreased connectivity between the left RN and the ipsilateral middle frontal gyrus. For the right RN, the comparison between the two groups revealed significantly decreased FC in the right superior parietal lobe and increased FC in the ipsilateral cerebellum (Fig. 1; Table 2). Additionally, patients with MwoA demonstrated significantly decreased FC between the right SN and some regions encompassing the right postcentral gyrus, left parietal lobule, and left superior frontal gyrus, whereas no significantly increased or decreased FC was observed between the left SN and other brain regions (Fig. 2; Table 3).

**Table 2:** Abnormal functional connectivity of the bilateral red nucleus in patients with MwoA compared with healthy controls

	Brain region	Peak MNI coordinates			Voxel size	Peak t score
		X	Y	Z		
L Red Nucleus	L Middle Frontal Gyrus	−18	6	48	293	−3.8345
R Red Nucleus	R superior parietal lobe	12	−60	63	245	−3.7167
	R Cerebellum	6	−54	−12	73	5.1646

Significance was determined at a voxel-level threshold (*P* < 0.01) with Gaussian random field theory correction at a cluster-level threshold (two-tailed, *P* < 0.05). *MwoA* Migraine without aura, *MNI* Montreal Neurological Institute

**Table 3:** Abnormal functional connectivity of the bilateral substantia nigra in patients with MwoA compared with healthy controls

	Brain region	Peak MNI coordinates			Voxel size	Peak t score
		X	Y	Z		
L Substantia Nigra	NaN					
R Substantia Nigra	R Postcentral Gyrus	36	−36	48	73	−4.4389
	L Parietal Lobule	−3	− 36	72	223	−5.0468
	L Superior Frontal Gyrus	−30	−3	57	75	− 4.3164

Significance was determined at a voxel-level threshold (*P* < 0.01) with Gaussian random field theory correction at a cluster-level threshold (two-tailed, *P* < 0.05). *MwoA* Migraine without aura, *MNI* Montreal Neurological Institute

### Correlation analysis

**Fig. 3:**
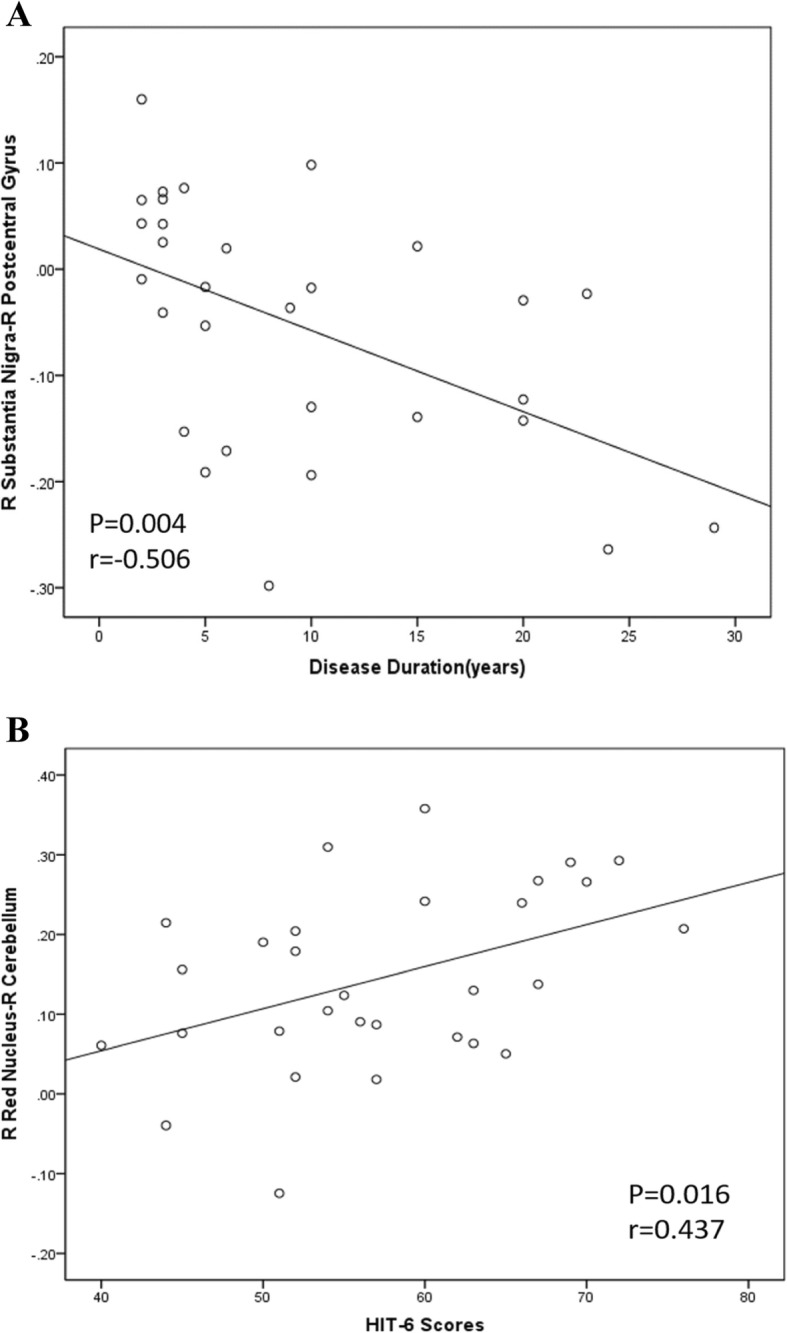
**a** 3BSignificant correlations between the brainstem structures’ functional connectivity and the characteristics of the patients with migraine without aura. Correlations between the functional connectivity of the right substantia nigra-right postcentral gyrus and disease duration (**a**). Correlations between the functional connectivity of the right red nucleus-right cerebellum and the Headache Impact Test-6 scores (**b**)

As exhibited in Fig. 3a and b, hypo-connectivity between the right SN and right postcentral gyrus was negatively correlated with disease duration (*r* = − 0.506, *P* = 0.004). Further analysis revealed that the increased connectivity of the right RN to the ipsilateral cerebellar lobes was positively correlated with the HIT-6 scores (*r* = 0.437, *P* = 0.016). No other significant linear correlations were observed between the FC of the left middle frontal gyrus, right superior parietal lobe, left parietal lobe, and superior frontal gyrus and the neuropsychological test results and attack frequency of patients with MwoA.

## Discussion

It has been suggested that rs-fMRI is a more sensitive technique for neuropsychological assessment. Previously, diagnosis of “structure normal” disease like depression and schizophrenia often relied on questionnaire, electroencephalogram, etc. [27]. rs-fMRI using Bold sequence reflected inherent abnormality and revealed more foudmental brain functional aleration along with grey/wihte matter structural change in patients with brain disease, injury or dysfunction [28, 29]. Current study showed that patients with MwoA had disrupted resting-state RN/SN FC with a set of brain regions including diverse functional areas. Available evidence supports malfunctioning of the RN and SN as an important factor for migraine [15–18]. Additionally, the RN and SN play crucial roles in a range of cognitive, motor, and memory controls. Therefore, functional connectivity changes in the RN and SN warrant further study in patients with MwoA. To our knowledge, this is the first examination of RN and SN functional connectivity in interictal MwoA.

Our study indicated that there is significantly decreased FC between the left RN and ipsilateral middle frontal gyrus. Areas within the frontal lobe are also part of the brain network involved in pain processing. In particular, the middle frontal gyrus, as part of the prefrontal cortex, is considered to be involved in the cognitive evaluation and modulation of pain [30]. One previous CSD tractography study with healthy controls revealed that the RN was sparsely connected with other cortical areas, including the middle frontal gyrus [11]. The middle frontal gyrus is reportedly involved in the attentional network. More specifically, the middle frontal gyrus has been proposed to contribute to both the dorsal (top-down) and ventral (bottom-up) attention networks [31, 32]. Based on a task-based resting-state fMRI study, Edina reported significant neural activation in response to fearful faces in the right middle frontal gyrus in patients with migraine relative to controls [33]. Activation of this region might be associated with emotional management in patients with migraine. Structural abnormalities have also been previously reported. A large cohort study showed that patients with MwoA had a thinner cortex bilaterally in the middle frontal gyrus than did healthy controls [34]. A study by Russo et al. showed that migraineurs had significant FC reduction within the right frontoparietal networks compared to healthy controls, and reduced connectivity in the middle frontal gyrus was negatively correlated with the pain intensity of migraine attacks [35]. Our result of altered FC between the left RN and ipsilateral middle frontal gyrus in interictal individuals with migraine is consistent with other researchers, indicating both functional and structural alteration in the middle frontal cortex as abnormal neuronal activation thresholds. Thus, impaired FC related to the middle frontal gyrus diminished the capacity for pain modulation and evaluation, which might underline the mechanism of irritable and impulsive reactions in patients with MwoA.

In the present resting-state functional study, we also detected lower connectivity between the right RN and right superior parietal lobe. However, contrary to this finding, patients during a spontaneous migraine attack exhibited increased FC between the right thalamus and several contralateral brain regions including the superior parietal lobe [36]. A previous study showed an increased gyrification index in the left superior parietal lobe in patients with MwoA compared with healthy controls [37]. In a previous positron emission tomography with computed tomography study, the migraine group had decreased glucose metabolism in several contralateral brain regions including the superior parietal lobe [38]. In fact, it has been proposed that the superior parietal lobe is involved in the sensory discrimination of pain information [30, 39]. Thus, impaired connectivity related to the superior parietal lobe may disrupt the pathway used to discriminate the sensory features of pain and induce hypersensitivity to pain stimuli in migraineurs.

Our data showed FC between the right SN and the contralateral superior frontal gyrus. The superior frontal gyrus may modulate the cortical and subcortical nociceptive pathways [40]. Gray matter volume reduction of the dorsolateral and medial parts of the superior frontal gyrus was observed at the 1-year follow-up in a group of patients with MwoA [41]. Functional changes in the frontal lobe have also been reported in other pain disorders. In a recent regional homogeneity (ReHo) study, Wang et al. [42] reported that patients with tension-type headache exhibited lower ReHo values in the superior frontal gyrus. MwoA may also involve superior frontal gyrus functional abnormalities as do other pain disorders.

Our results also suggested significantly reduced FC between the right SN and right postcentral gyrus and left parietal lobule. The altered FC between the right SN and homolateral postcentral gyrus was negatively associated with migraine duration. The postcentral gyrus, which is part of the primary somatosensory cortex, predominantly participates in sensory-discriminative pain processing. It showed extensive connections running from the primary somatosensory area to the homolateral SN in a previous CSD tractography study [14]. The reduced connectivity between the right SN and the homolateral somatosensory area may be associated with disrupted projections and modulation of perception and pain. fMRI studies have revealed activation of postcentral regions in response to painful stimulation [43, 44]. Hypoperfusion in the postcentral gyrus was found in migraineurs in the interictal state using dynamic susceptibility contrast MRI [45]. Our findings of reduced FC within the somatosensory area are in line with those of other studies showing decreased amplitude of low-frequency fluctuation [46] and cortical thinning [47] within the postcentral gyrus in migraineurs. Furthermore, the altered FC between the right SN and homolateral postcentral gyrus was negatively associated with migraine duration. Abnormalities of postcentral gyrus FC may comprise part of the disrupted networks associated with the aberrant structures and function processing observed in migraine. We speculate that nociceptive input modifies the functional patterns of the somatosensory cortex and that these changes may account for the functional impairments in MwoA.

By contrast, we found significantly increased connectivity between the right RN and the ipsilateral cerebellum. Interestingly, this increased connectivity was positively related to the HIT-6 scores. Recent functional imaging studies have found robust FC between the cerebellum and many of the known migraine-related areas in the brainstem, midbrain, and cortex, indicating that the cerebellum may have an important role in pain processing [47, 48]. The cerebellum isin communication with the RN, forming a cerebello-thalamo-pre-supplementary motor area-RN feedback loop, which might be implicated in concentration-related optimization for upcoming motor performance [49]. The cerebellum also has anatomical connections with multiple areas of the frontal cortex and limbic system, which are critical for its involvement in cognitive and pain processing. Our result of increased FC of the cerebellum is in agreement with those of several previous studies. Altered low-frequency fluctuations in amplitude were observed in the cerebellum of migraineurs in the interictal period [46]. fMRI of patients during a migraine episode evoked by olfactory stimuli showed significantly increased activity compared to interictally-recorded activity prior to the migraine [50]. Increased cerebellar activity has been shown in response to trigeminal noxious heat stimulation in migraineurs compared with healthy controls [51]. This increased connectivity may reflect a long-time compensatory mechanism, and the impaired neurons require heightened connectivity to create the same signal. In addition, the increased connectivity between the right RN and the ipsilateral cerebellum was positively related to the HIT-6 scores of the migraineurs in the present study. We speculate that these pain-related brain regions provided feedback to the brainstem as pain intensity increases and might underline the mechanism of the hypersensitive response to external stimuli in migraine.

Our study has several potential limitations. First, the sample size was relatively small and does not suffice for generalization of the study findings; there is need for large-scale clinical validation. Second, this study did not enroll typical patients with MwA and a chronic-pain control group, which could establish the specificity of the findings to migraine. Third, our study were limited to a heterogeneous population of patients with MwoA. Chronic migraineurs were not categorized out from whole migraineurs due to small case number(4 with chronic migraine and 26 with episodic migraine). The ICHD-3β criteria for chronic migraine diagnosis required headache at least 15 days/month for least 3 months, it was restrictive to include enough chronic migraineurs compared with Expert Opinion criteria published in 2014 [52]. Fourth, the seed used in this study covered the whole RN and SN; however, the RN was subdivided into the large and small cell portions, and the SN was subdivided into the dorsal pars compacta and pars reticulata. Different parts may have their own distinct impact; therefore, more accurately defined anatomical parcellation of the RN and SN may influence the final seed-based FC maps, which may improve the specificity of the analysis. Finally, the analytical method of FC has intrinsic limitations in terms of anatomical connections and the direction of information flow from one area to another. These issues will be addressed in subsequent studies.

## Conclusion

In conclusion, our results suggested that patients with MwoA have disruption in their RN and SN resting-state networks. This may be associated with functional impairments in pain processing that play a key role in the clinical characteristics of MwoA.

## Data Availability

All data and materials generated in this study are available upon request.
